# A randomized placebo-controlled trial to investigate the effect of lactolycopene on semen quality in healthy males

**DOI:** 10.1007/s00394-019-02091-5

**Published:** 2019-10-08

**Authors:** Elizabeth A. Williams, Madeleine Parker, Aisling Robinson, Sophie Pitt, Allan A. Pacey

**Affiliations:** Department of Oncology and Metabolism, Faculty of Medicine Dentistry and Health, Medical School, The University of Sheffield, Beech Hill Road, Sheffield, S10 2RX UK

**Keywords:** Lactolycopene, Sperm motility, Sperm morphology, Fertility

## Abstract

**Purpose:**

Poor sperm quality is a major contributor to infertility in heterosexual couples, but at present there are few empirical therapies. Several studies have examined the role of dietary factors and data from randomized controlled trials suggest that oral antioxidant therapy can improve some sperm parameters. Health benefits of lycopene supplementation have been proposed for a variety of health conditions and here we examine whether it can help improve sperm quality. This study aimed to investigate the effect of 14 mg daily lactolycopene for 12 weeks on semen quality in healthy men.

**Methods:**

Sixty healthy male participants were recruited and randomized to this double-blind, placebo-controlled parallel study and received either 14 mg/d lactolycopene or a placebo for 12 weeks. The primary endpoint was a change in motile sperm concentration. Secondary endpoints were all other aspects of sperm quality, including the level of sperm DNA damage.

**Results:**

Fifty-six men completed the intervention and the level of plasma lycopene was significantly increased in the men randomized to receive lycopene supplementation. There was no significant change in the primary endpoint (motile sperm concentration) post-intervention (*p* = 0.058). However, the proportion of fast progressive sperm (*p* = 0.006) and sperm with normal morphology (*p* < 0.001) did improve significantly in response to lactolycopene intervention.

**Conclusions:**

Supplementation with 14 mg/d lactolycopene improves sperm motility and morphology in young healthy men.

**Clinical Trial Registry number and website:**

ISRCTN33248724 http://www.isrctn.com/ISRCTN33248724

## Introduction

Current estimates suggest that infertility affects up to 16.7% of heterosexual couples in developed countries [1] and it is generally recognized that male sub-fertility plays a contributory role in up to 50% of cases [2]. Typically, fertility problems in the male manifest themselves as ejaculates containing too few sperm (oligozoospermia), or sperm that swim poorly (asthenozoospermia), or sperm with poor size and shape (teratozoospermia) or a combination of all three [3].

Unfortunately, for most sub-fertile men, there are few empirical therapies to improve poor sperm quality [4]. As such, for most men, healthcare professionals can only focus on delivering general advice to highlight the known lifestyle risks for poor sperm quality. For example, epidemiological studies have shown how certain occupations [5, 6], the wearing of tight underwear [7, 8], tobacco smoking [9], high body mass index (BMI) [10] and recreational drug use [11] are each associated with aspects of poor sperm quality.

Similarly, there is some evidence that dietary factors are also correlated with sperm quality, for example, data on average daily nutrient intake from food and supplements obtained from self-administered food frequency questionnaires found that higher antioxidant intake was associated with higher sperm concentration and motility [12]. However, although over 25 randomized controlled trials to test the effect of oral antioxidant supplementation (single or combined) versus placebo on semen quality have been conducted, the results are largely inconsistent and inconclusive due to their poor quality and high heterogeneity of design [13].

Of the many oral antioxidants examined for their effects on semen quality, a recent systematic review [14] concluded that diets rich in vitamin E, vitamin C, β-carotene, selenium, zinc, cryptoxanthin and lycopene were inversely associated with poor sperm quality. Of these, lycopene has had renewed interest in other aspects of male reproductive health. For example, in a recent study of nearly 50,000 male health professionals, dietary lycopene was associated with a reduced risk of lethal prostate cancer [15] and a dose–response meta-analysis of 34 studies suggested that the risk was reduced by 3% per 1 mg/day increment of dietary lycopene intake [16]. There have been only a few trials to date that have directly considered the effect of lycopene on semen parameters (reviewed in [17]). A few human studies include an open-label study of 30 men with idiopathic infertility allocated to 4 mg lycopene daily for 12 weeks, where improvements were seen in sperm concentration, motility and morphology in a high proportion of the men [18]. Another study of 50 oligoasthenozoospermic men prescribed 8 mg of lycopene each day for an indefinite period reported the optimisation of sperm parameters in 36% of them [19]. A more recent study reported a transient improvement in sperm motility after 6 weeks in infertile men randomized to a 12-week intervention of tomato juice, providing 30 mg lycopene each day, compared to a no-intervention control [20]. However, the complexity of the tomato juice food matrix, which also provided vitamin C and E, makes it difficult to attribute the apparent improvement to lycopene alone. The lack of an effective placebo control group in each of these studies limits their interpretation and generalisability and highlights the need for a randomized placebo control trial.

Therefore, given the clinical interest and potential for dietary supplements to improve semen quality, as well as the renewed interest in antioxidants and specifically lycopene, we reasoned that a randomized controlled trial of lycopene supplementation was warranted. In this paper, we, therefore, report the outcome of a double-blind, placebo-controlled randomized trial to investigate the effect of 3 months of oral lactolycopene supplementation on the semen quality of healthy male volunteers.

## Methods

Healthy male volunteers were recruited from the Sheffield city region in April and May 2016 using a brief campaign conducted by email, social media and leaflets targeted at (but not limited to) staff and students at the University of Sheffield (UK). Potential volunteers registered their interest in participating in the study by e-mail and subsequently underwent a telephone eligibility screening. Men were excluded if they had previous testicular surgery, existing or previous cancer, or if they had a known allergy to tomato, whey protein or soy derivatives. Otherwise, they were sent an information sheet and invited to an enrolment visit if they were (i) aged between 18 and 30 years; (ii) lived within 1 h of the Royal Hallamshire Hospital, Sheffield; and (iii) were planning to live in the Sheffield city region for the duration of the study. The primary outcome of the study was motile sperm concentration, and secondary outcomes were sperm volume, concentration, motility, morphology, DNA damage and plasma lycopene concentration measured at baseline and end of intervention (week 12). There were no robust data on which to base a sample size calculation. The data provided by Gupta et al. (2002) [18] could not be used for a formal power calculation due to their reporting method. Thirty infertile men were recruited in that study, but data were only reported for the men that showed an improvement for one of the sperm parameters (*n* = 20 for sperm concentration) and means (± SD) were not provided for the whole population. Nevertheless, this paper did provide an indication of the likely sample size required, and on this basis, we aimed to recruit 30 participants per treatment arm.

At the enrollment visit, men were given the opportunity to ask further questions and, if they agreed to participate, invited to sign a consent form. Then basic demographic variables were recorded, and men completed a brief questionnaire about their lifestyle choices (e.g. smoking habits, alcohol consumption and choice of underwear). In addition, their height and weight were recorded, and their BMI was calculated in kg/m^2^. A date was then agreed for them to start the study and attend the follow-up visits in weeks 6 and 12 of the study, respectively.

The study design was a randomized, double-blind, placebo-controlled trial and at the first visit, men were randomly allocated to either treatment or placebo groups using a block randomisation schedule in blocks of four. The randomisation code was created using a randomization generator (www.randomization.com) and held by a third party until all analysis was complete, and all capsules were identically boxed to ensure both the researchers and volunteers were blind to the allocation. The lactolycopene supplement was manufactured and provided by Cambridge Nutraceuticals Ltd. (Cambridge, UK) who also provided the placebo. The supplement was supplied in capsule form and provided 7 mg lycopene per capsule within a whey protein matrix formulated to enhance lycopene absorption [21]. The placebo capsules were identical in appearance but contained microcrystalline cellulose. Participants were instructed to take two capsules per day with water (one in the morning and one in the evening) for 12 weeks (May to September 2016). Compliance was calculated on the basis of number of capsules returned at the end of the study as a percentage of the number of capsules expected to have been consumed during the intervention period.

All participants were asked to provide a semen sample (after 3 days of sexual abstinence) at the first (baseline) visit and also at the follow-up appointment arranged in week 6 and at the end of the study (week 12). In the 7 days prior to each visit, men were supplied with a pre-weighed sterile container (Sarstedt, Leicester, UK), reminded of the abstinence period and asked to record the time of production and deliver the semen sample to the laboratory within 1 hour. Upon arrival, the sample was analysed according to World Health Organisation (2010) methods and, in addition, sperm motility was assessed using version 5.0 of the Sperm Class Analyzer (Microptic SL, Barcelona, Spain) attached to a Microtec LM-2 Microscope (Mazurek Optical Services Ltd, Southam, UK) via a Basler A312fc camera (Basler AG, Ahrensburg, Germany) [22]. This assigned each sperm observed in one of four motility grades: (i) fast-progressive (≥ 25 µm per second), (ii) slow-progressive (≥ 5 and < 25 µm per second), (iii) non-progressive (< 5 µm per second), and (iv) immotile (0 µm per second). Slides were also prepared for the assessment of sperm DNA fragmentation by TUNEL according to the methods published previously [23].

At each study visit, 5 ml of non-fasting blood was collected by venepuncture into EDTA tubes (BD Bioscience, Wokingham, UK). Blood samples were centrifuged immediately (400 g for 10 min) to separate the plasma and red blood cells. Plasma was stored in light-protected Eppendorf tubes (BD Bioscience, Wokingham, UK) and frozen at − 70 °C prior to analysis. Plasma lycopene concentrations were analysed by reverse-phase HPLC with ultraviolet detection [24] at the Department of Clinical Biochemistry, Glasgow Royal Infirmary.

Dietary intake was assessed at baseline and at the end of the intervention using a 4-day food diary (including 1 weekend day) as previously outlined [25]. Food portion sizes were verified by an interview with the researcher (AR) using the Ministry of Agriculture Food and Fisheries food atlas [26]. The 4-day estimated food diary was analysed for macronutrient and micronutrient content using WinDiets Research software (version 2016; Robert Gordon University, Aberdeen, United Kingdom).

All statistical analyses were performed in SPSS (Version 25). Data were checked for normality using the Shapiro–Wilk test. Baseline differences between the groups were examined using unpaired *t* tests and Mann–Whitney *U* tests as appropriate. Within the group, changes from baseline were analysed using paired *t* tests and Wilcoxon signed-rank test. Categorical data were analysed using Chi-squared analysis. Many of the nutrient variables were not normally distributed and so non-parametric tests were used to analyse the data.

Ethical approval for the study (including the methods of enrolment) was obtained from the University of Sheffield’s Medical School Research Ethics Committee (SMBRER008135). The trial was registered at http://www.isrctn.com/ as ISRCTN33248724 and was conducted in accordance with the Declaration of Helsinki. Participants who completed the study received a £75 voucher to compensate them for their time and effort and to cover travel expenses.

## Results

**Fig. 1:**
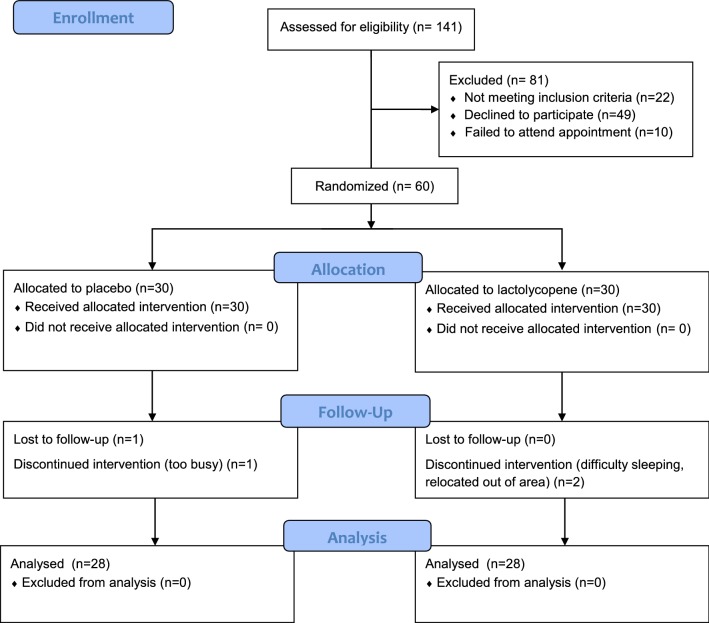
Consort flow chart of study. *CONSORT* consolidated standards of reporting trials

A total of 141 men responded to the recruitment campaign and expressed their interest in participating in the study. However, after being sent the information sheet, 39 decided not to continue and, after the initial telephone interview, 22 did not meet the inclusion criteria (see Fig. 1). Therefore, 80 men were invited to participate of which 70 signed a consent form at the enrolment visit and 60 attended the first appointment and provided a baseline semen analysis and blood sample. Four men withdrew from the study before the end, two from each arm of the study. No adverse events were reported by any of the participants. The duration of the intervention was similar in both treatment arms with a median duration of 84 days in both treatment arms (Table 1). Fifty of the 56 men who completed the trial returned their unused capsules at the end of the study. Compliance with the intervention was good and similar in both treatment arms, with median compliance of 97.6% and 94.3% in the placebo and lactolycopene arms, respectively (Table 1).

**Table 1:** Baseline characteristics, duration of intervention and compliance with the intervention in the participants who completed the study

Characteristic	All (*n* = 56)	Placebo (*n* = 28)	Lactolycopene (*n* = 28)	*p*
Age (years)	23.3 ± 2.89	23.3 ± 2.58	23.4 ± 3.22	0.855
Smokers (*n*)	8	4	4	1.0
Street drug use				
Yes [*n* (%)]	4 (7%)	2 (7%)	2 (7%)	1.0
No [*n* (%)]	52 (93%)	26 (93%)	26 (93%)	
Underwear type (*n*)				
Tight	34	17	17	1.0
Loose	22	11	11	
BMI (kg/m^2^)	24.4 ± 3.18	23.5 ± 3.11	25.2 ± 3.08	0.049
Alcohol consumption units/w [median (min–max)]^a^	8 (0–40)	7 (0–28)	10 (0–40)	0.136
Plasma lycopene (µmol/l)^b^	0.666 ± 0.255	0.689 ± 0.286	0.645 ± 0.226	0.532
Number of intervention days [median (IQR)]	84 (1.0)	84 (1.0)	84 (1.75)	0.545
	*(n = 50)*	*(n = 26)*	*(n = 24)*	
Percent compliance [median (IQR)]^c^	95.8 (9.23)	97.6 (8.48)	94.3 (10.57)	0.219

Mean ± SD (all such values) unless otherwise stated *p*: independent sample *t* test for continuous variables unless otherwise stated; Pearson’s Chi-square test for categorical variables

^a^Non-parametric Mann–Whitney *U*

^b^2 of the participants in the placebo arm had missing plasma lycopene at baseline

^c^Compliance was calculated as the total number of capsules returned as a percentage of the number of capsules expected to be consumed during the intervention period

Table 1 also shows the demographic characteristics of the 56 men who completed the study. Briefly, the mean ± SD age of the participants was 23.3 ± 2.89 years (range 19–30 years) and there was no significant difference between those randomized to placebo or lactolycopene, nor was there a significant difference between the proportion of smokers, street drug use, or choice of underwear, in either group. However, the mean ± SD BMI of men randomized to receive lactolycopene was significantly higher than those randomized to placebo (25.2 ± 3.08 vs 23.5 ± 3.11; *p* = 0.049). There was a wide range in habitual self-reported alcohol intake in the population with intakes ranging from 0 to 40 units per week, but the proportion of no/moderate and high consumers was similar in both treatment arms. Critically, there was no difference in plasma lycopene levels in either group at the start of the study.

Table 2 shows the semen quality data at baseline and shows the various measures were well balanced with no statistical differences between men taking lactolycopene versus placebo for any semen variable. Table 3 shows the levels of plasma lycopene, semen quality and clinical classification at the beginning and the end of the intervention. Briefly, whilst there was no change in the primary outcome measure of motile sperm concentration in either group, in the lactolycopene arm there was a significant increase in the percent of fast-progressive sperm (10.6 ± 8.75 vs 14.76 ± 10.29; *p* = 0.006), the percent of morphologically normal sperm (7.5 ± 5.49 vs 13.5 ± 4.90; *p* < 0.001) and in the plasma lycopene concentration (0.645 ± 0.226 vs 0.751 ± 0.221 µmol/l; *p* = 0.020) when the baseline measurement was compared to that obtained in week 12. In contrast, the only change in the placebo arm of the study was a statistical decrease in the percent of non-progressive sperm (24.6 ± 6.67 vs 21.4 ± 6.53; *p* = 0.002). Over the 12 weeks of study, there was no change in the percent of sperm with DNA damage. There was no significant difference at week 12 between the lactolycopene and placebo treatment arms for any of the semen quality parameters measured (*p* values not shown in table, all exceed 0.05; NS).

**Table 2:** Baseline semen quality (mean ± SD) of men randomized to receive lactolycopene or placebo

	All *n* = 56	Placebo *n* = 28	Lactolycopene *n* = 28	*p*
*(a) Semen quality*				
Abstinence (d)^a^	3.2 ± 0.82	3.1 ± 0.54	3.3 ± 1.02	0.248
Semen volume	4.1 ± 1.66	4.0 ± 1.57	4.2 ± 1.77	0.635
Sperm concentration (× 10^6^/ml)^a^	63.4 ± 50.79	56.8 ± 43.24	70.0 ± 57.40	0.523
Percent motility (%)	61.1 ± 23.78	62.8 ± 22.69	59.4 ± 25.13	0.597
Fast progressive (%)	11.2 ± 7.76	11.8 ± 6.96	10.6 ± 8.57	0.551
Slow progressive (%)	24.9 ± 14.51	26.3 ± 14.74	23.5 ± 14.40	0.474
Non-progressive (%)	25.0 ± 7.63	24.6 ± 6.67	25.3 ± 8.58	0.752
Immotile (%)	38.9 ± 23.79	37.2 ± 22.68	40.6 ± 25.14	0.598
Motile sperm concentration (× 10^6^/ml)^b^	27.0 ± 31.03	26.2 ± 31.68	27.8 ± 30.93	0.597
Sperm with normal morphology (%)^a^	8.3 ± 5.40	9.1 ± 5.28	7.5 ± 5.48	0.144
DNA damage (%)^a^	1.5 ± 1.56	1.4 ± 1.65	1.6 ± 1.48	0.366
*(b) Clinical classification*^c^				
Normozoospermic (*n*)	25	14	11	0.420
Other (*n*)	31	14	17	

*p*: independent sample* t* test or ^a^Non-parametric Mann–Whitney* U* for continuous variables

^b^Expressed as the concentration of fast-progressive and slow-progressive sperm

^c^Pearson chi squared

**Table 3:** Plasma lycopene and semen quality (mean ± SD) and clinical classification (*n*) of baseline versus week 12 for men randomized to receive placebo or lactolycopene supplementation

	Placebo arm (*n* = 28^a^)			Lactolycopene arm (*n* = 28^a^)		
Variable	Baseline	Week 12	*p*	Baseline	Week 12	*p*
Plasma lycopene (µmol/l)	0.689 ± 0.286	0.719 ± 0.306	0.563	0.645 ± 0.226	0.751 ± 0.221	**0.020**
Semen quality:						
Abstinence (d)^b^	3.1 ± 0.54	3.5 ± 1.11	0.102	3.3 ± 1.02	4.4 ± 5.11	0.393
Semen volume (ml)	4.0 ± 1.57	4.1 ± 2.56	0.873	4.2 ± 1.77	3.7 ± 1.57	0.116
Sperm concentration (× 10^6^/ml)^b^	56.8 ± 43.24	62.8 ± 55.10	0.569	70.0 ± 57.40	67.6 ± 52.47	0.81
% Motility	62.8 ± 22.69	59.3 ± 26.53	0.332	59.4 ± 25.13	64.3 ± 21.79	0.205
% Fast progressive	11.8 ± 6.96	12.5 ± 7.90	0.696	10.6 ± 8.57	14.76 ± 10.29	**0.006**
% Slow progressive	26.3 ± 14.74	25.5 ± 17.35	0.690	23.5 ± 14.40	24.92 ± 11.43	0.491
% Non-progressive	24.6 ± 6.67	21.4 ± 6.53	**0.002**	25.3 ± 8.58	24.6 ± 7.33	0.649
% Immotile	37.2 ± 22.68	40.7 ± 26.53	0.333	40.6 ± 25.14	35.7 ± 21.79	0.206
Motile sperm concentration (× 10^6^/ml)^b,c^	26.2 ± 31.7	29.6 ± 37.5	0.509	27.8 ± 30.9	31.3 ± 30.3	0.058
Sperm with normal morphology (%)^b^	9.1 ± 5.28	11.3 ± 6.6	0.124	7.5 ± 5.49	13.5 ± 4.90	**<0.001**
% DNA damage^b^	1.4 ± 1.65	1.5 ± 2.25	0.366	1.6 ± 1.49	2.1 ± 3.21	0.476
Clinical classification^d^						
Normozoospermic (*n*)	14	13	0.763	11	18	0.052
Other (*n*)	14	15		17	9	

^a^2 of the participants in the placebo arm had missing plasma lycopene at baseline and 1 had missing plasma lycopene at week 12

*p*: paired* t* test or ^b^Wilcoxon signed-rank test for continuous variables

^c^Expressed as the concentration of fast-progressive and slow-progressive sperm

^d^Cochran Q test; 1 of the participants in the lactolycopene arm had missing sperm motility and motile sperm concentration data at week 12

Energy and nutrient intake at baseline and at the end of intervention for the two arms of the study are shown in Table 4. There were no significant differences between the groups at baseline and no significant change in dietary intake in response to treatment in either arm. Dietary intake was not significantly different between treatment arms at week 12 (*p* values not shown in table, all exceed 0.05; NS).

**Table 4:** Energy and nutrient intake^a^ at baseline versus week 12 for men randomized to receive placebo or lactolycopene supplementation

	Placebo arm (*n* = 28)			Lactolycopene arm (*n* = 28)		
Variable	Baseline^b^	Week 12^b^	*p*^c^	Baseline^b^	Week 12^b^	*p*^c^
Energy (kJ/d)	9327 (2609.8)	9871 (3290.5)	0.964	9614 (3737.3)	10,997 (6425.8)	0.265
Energy (kcal/d)	2178 (755.0)	2358 (779.3)	0.982	2252 (854.5)	2617 (1525.0)	0.295
Protein (g/d)	91.5 (53.2)	92.8 (33.3)	0.425	96.0 (46.5)	107.3 (80.8)	0.412
Fat (g/d)	88.5 (56.3)	89.8 (44.7)	0.982	93.1 (47.6)	93.3 (63.9)	0.295
Saturated fat (g/d)	31.4 (16.8)	28.4 (17.6)	0.554	28.9 (18.5)	35.6 (23.0)	0.172
Carbohydrate (g)	254 (126.6)	233 (89.3)	0.767	245 (70.5)	247.8 (156.1)	0.767
Dietary fibre (g/d)	17.7 (9.2)	17.2 (11.4)	0.539	20.0 (10.9)	18.1 (13.6)	0.973
Alcohol (g/d)	8.95 (23.1)	4.6 (23.0)	0.931	6.7 (28.4)	5.4 (23.4)	0.615
Vitamin A (µg/d)	556 (490.8)	757 (55.8)	0.452	685 (886.5)	617 (462.8)	0.699
Vitamin C (mg/d)	70.7 (74.5)	81 (69.1)	0.480	72.2 (72.8)	65.1 (96.3)	0.227
Vitamin E (mg/d)	11.3 (6.21)	10.3 (6.76)	0.909	11.4 (5.77)	10.2 (6.53)	0.716
Zinc (mg/d)	10.8 (5)	9.8 (4)	0.531	10.4 (6.5)	12.7 (5.3)	0.585
Selenium (µg/d)	62.5 (37.5)	54.0 (31.8)	0.344	51.5 (32.8)	69.5 (49.0)	0.096
Retinol (µg/d)	310 (168.5)	296 (268.3)	0.649	305 (351.5)	337 (203.3)	0.327
Carotene (µg/d)	1507 (2823.5)	1500 (3789.8)	0.820	1844 (3691.8)	1291 (2163.5)	0.509

^a^Energy and nutrients derived from the diet and non-intervention supplements

^b^Non-parametric Mann–Whitney *U* was used to compare dietary intake between the groups at baseline and end of intervention. No significant differences were found

^c^Wilcoxon signed-rank test was used to examine within group change from baseline. Data are presented as medians (IQR)

## Discussion

Lycopene is a carotenoid found in high concentrations in tomatoes, tomato products and other red-pigmented foods which has received considerable interest in recent years as a potential therapeutic agent for a variety of health conditions [27]. However, the bioavailability of lycopene from fresh tomatoes is low, but this is enhanced by processing, heating and co-delivery of tomatoes with oil [28]. As such, this study used the nutritional supplement lactolycopene, the main ingredient of which is lycopene embedded in a whey protein matrix for enhanced intestinal absorption. The 14 mg/d of lactolycopene supplied to the men in this study is equivalent to consuming 2 kg of cooked tomatoes or 2 tablespoons of concentrated tomato puree each day, and so represented a sizable increase in the lycopene intake of the study participants.

For this study, we chose to enrol as our study participants young healthy volunteers with no known fertility issues. However, this was done with the knowledge that a sizeable proportion of them would have poor sperm quality at the outset [29] but since they were less likely to have tested their fertility and they were not actively trying to conceive, they would be blind to their own semen quality (unlike fertility patients attending a clinic) and, therefore, unlikely to make other adjustments to their lifestyle to enhance fertility. It is noteworthy that at the outset of the study, 31 of the 56 men (see Table 3) did not have normal semen parameters (normozoospermia), suggesting that our original assumption was correct.

For the primary outcome measure, we chose to use the concentration of motile sperm since this has been shown to be the variable obtained from semen analysis most likely to be associated with the probability of conception [30]. However, whilst this was not statistically improved after lycopene supplementation (*p* = 0.058) two other measures of sperm quality were significantly altered: (i) the proportion of fast-progressive sperm (*p* = 0.006) and (ii) the proportion of sperm with normal morphology (*p* < 0.001). The direction of both these changes is positive, but it is impossible to say how they might impact chances of natural pregnancy or the choice of assisted reproduction treatment (ART) had our participants been attending an infertility clinic. In theory, both are clinical improvements in sperm quality and would presumably be welcomed by infertile men. Whilst many measures of sperm motility [31, 32] and morphology [33] are difficult to make, we used computer-assisted sperm analysis for both measures, which provides a level of objectivity; furthermore, the sperm quality measures were performed blind to the allocation of each participant. In comparison to the changes observed in the men randomized to lycopene supplementation, the only statistically significant change observed in the placebo group was a reduction in the percent of non-progressive sperm (*p* = 0.002). We are unable to explain this observation but conclude that it is unlikely to be clinically significant since it was only accompanied by a non-significant increase in the percent non-motile sperm, and sperm with these motility characteristics are unlikely to participate in natural conception [2]. Interestingly, we saw no change in sperm DNA damage, but given the ongoing controversy about its measurement and interpretation [34] this is perhaps not surprising.

The 12-week duration of the study was chosen to increase plasma lycopene levels across the full (~ 70 day) window required to make (and ejaculate) new sperm [35] because we did not want to make any assumptions about when lycopene supplementation might be most beneficial. Therefore, it is not possible to conclude whether there is an optimum duration of lycopene supplementation that would give the same results as those reported here without undertaking further study.

Similarly, the choice of lactolycopene dose was a pragmatic one based on the availability of the supplement from the supplier, and the use of similar lycopene dosages and durations in clinical trials of cardiovascular disease [36]. So, again, we are unable to comment whether different doses of the supplement to the ones used here may lead to the same results. However, it is noteworthy that the results we describe are similar to those previously reported in an open-label study showing improvements in sperm morphology and motility using 4 mg of daily lycopene supplementation for 3 months [18]. The daily dose of 14 mg lycopene was certainly able to significantly increase the plasma lycopene level in our study participants (see Table 3) compared to those randomized to placebo. However, whether this is the optimum level to maximize any effects on sperm quality measures remains to be established.

The underlying biological mechanism of action whereby lycopene exerts an effect on the sperm is currently unknown. The antioxidant properties of lycopene have been the primary focus of mechanistic investigation of the action of lycopene on idiopathic male infertility to date [17]. Oxidative stress is believed to play a role in the pathogenesis of idiopathic male infertility and although reactive oxygen species play an important role in normal sperm function, disequilibrium of reactive oxygen species and antioxidant defence appears to be detrimental [37]. While it is well known that sperm are very vulnerable to damage by free radicals [38], we cannot assume that the beneficial effects of lycopene we observed are because of its antioxidant properties as we did not make any relevant measurements of oxidative stress in biological fluids such as seminal plasma. However, an antioxidant role for lycopene is a plausible hypothesis.

The study reported here had a modest sample size and is not without its limitations. First, to increase the rates of compliance by the study participants, we allowed them to produce their samples at home rather than in a clinic and this would have inevitably led to a delay in undertaking some of the measurements we have reported here. However, the World Health Organisation Laboratory Manual for the examination and processing of human semen [3] recommends that all measurements be performed within 1 h of sample production and others [39] have argued that specimen collection at home is not detrimental as long as samples are delivered in a timely manner and motility measurements are done at the correct temperature. In this study, the median delay in processing the samples was 58.5 min (range 35–115 min) and was not different between those men randomized to lycopene vs placebo. Furthermore, all motility measurements were undertaken at 37 °C as per WHO (2010) recommendations. Second, it is known that BMI is related to aspects of semen quality [10] and although the majority of the study participants had a normal BMI, a high proportion (45%) fell in the overweight or obese category. This may reflect a high muscle mass rather than a high fat mass since a proportion of the men were recruited via University Sports clubs. Interestingly, the BMI of men randomized to lycopene was significantly higher (*p* = 0.049) compared to those randomized to placebo (Table 1) which suggests that the observed improvement in semen quality in these men could be more remarkable than in men with a lower BMI. However, the difference in BMI is small and probably biologically insignificant, although in future studies it could be useful to consider undertaking an analysis of body composition using bioelectrical impendence [40] for a more accurate assessment of percentage body fat.

In conclusion, we report a modest but statistically significant improvement in the semen quality of healthy young men randomized to receive 14 mg lactolycopene per day for 12 weeks. Whilst the study demonstrates improvements in some measures of sperm quality in response to lycopene supplementation, the clinical impact on fertility and the chances of pregnancy, and live birth are unknown. Future studies should focus on men from infertile partnerships to determine not only the optimum dose and timing of lycopene both to improve sperm quality but also whether this enhances pregnancy outcome for these couples.
